# Effect of Intradialytic Oral Nutritional Supplementation with or without Exercise Improves Muscle Mass Quality and Physical Function in Hemodialysis Patients: A Pilot Study

**DOI:** 10.3390/nu14142946

**Published:** 2022-07-19

**Authors:** Geovana Martin-Alemañy, Monserrat Perez-Navarro, Kenneth R. Wilund, Gloria García-Villalobos, Irma Gómez-Guerrero, Guillermo Cantú-Quintanilla, Miguel Angel Reyes-Caldelas, Angeles Espinosa-Cuevas, Galileo Escobedo, Mara Medeiros, Paul N. Bennett, Rafael Valdez-Ortiz

**Affiliations:** 1 Nephrology Service, Hospital General de México “Dr. Eduardo Liceaga”, Mexico City 06726, Mexico; lucymonsepn@yahoo.com.mx (M.P.-N.); gggdharma@yahoo.com (G.G.-V.); mimigogue19@hotmail.com (I.G.-G.); 2 Department of Kinesiology and Community Health, University of Illinois, Urbana, IL 61820, USA; kwilund@illinois.edu; 3 Division of Nutritional Sciences, University of Illinois, Urbana, IL 61820, USA; 4 Facultad de Medicina, Universidad Panamericana, Mexico City 03920, Mexico; gcantu@up.edu.mx; 5 Department of Radiology and Image, Hospital General de México “Dr. Eduardo Liceaga”, Mexico City 06726, Mexico; reyescaldelasmiguelangel@gmail.com; 6 Departamento de Nefrología y Metabolismo Mineral, Instituto Nacional de Ciencias Médicas y Nutrición Salvador Zubirán, Mexico City 14080, Mexico; angespinosac@gmail.com; 7 Health Care Department, Universidad Autónoma Metropolitana, Mexico City 14387, Mexico; 8 Laboratorio de Proteómica, Dirección de Investigación, Hospital General de Mexico “Dr. Eduardo Liceaga”, Mexico City 06726, Mexico; gescobedo@unam.mx; 9 Unidad de Investigación y Diagnóstico en Nefrología y Metabolismo Mineral Óseo, Hospital Infantil de Mexico Federico Gómez, Mexico City 06720, Mexico; medeiromara@gmail.com; 10 Clinical and Health Sciences, University of South Australia, Adelaide 5000, Australia; paul.bennett@unisa.edu.au

**Keywords:** hemodialysis oral nutritional supplementation, aerobic exercise, anaerobic exercise, muscle mass, physical function

## Abstract

Background: Oral nutritional supplementation (ONS) with or without exercise (EX) could improve muscle mass (MM) in chronic kidney disease. Methods: Patients were randomized into two groups: (1) ONS and (2) ONS + EX. Thigh muscle area (cm^2^) and intramuscular lipid content via attenuation were evaluated at baseline and 6 months with computed tomography (CT) to measure MM quantity and quality. Physical function was measured by six-minute walk test (6 MWT), gait speed, handgrip strength (HGS), and Time Up and Go test (TUG) at baseline and 3 and 6 months. Results: The ONS group (*n*= 14) showed statistically significant improvement in gait speed and HGS; ONS + EX group (*n* = 10) showed differences in gait speed, in 6 MWT, and HGS. In the ANOVA (3 times × 2 groups), no differences were observed between groups. Greater effect sizes in favor to ONS + EX group were observed in the 6 MWT (d = 1.02) and TUG test (d = 0.63). Muscle quality at six months revealed a significant trend in favor of the EX-group (*p* = 0.054). Conclusions: Both groups had improved physical function, and greater effect sizes were seen in the ONS + EX group for the 6 MWT and TUG test. Neither MM quantity or quality was improved in either group.

## 1. Introduction

Skeletal muscle is one of the major tissues affected by chronic kidney disease (CKD) [1]. It is well known that patients undergoing chronic hemodialysis (HD) experience loss of muscle mass (MM), muscle strength [2], and physical function (PF) [3] due to many factors such as the dialysis procedure which induce a catabolic state, insufficient food intake, multiple endocrine disorders, persistent inflammation, acidosis, and physical inactivity, among others [4]. The reduction in MM and PF and poor nutritional status are directly associated with premature death, hospitalizations, frailty, and disability [1,5,6,7,8,9,10]. The improvement of both domains of MM (muscle size and quality) is important for dialysis patients because both are strong predictors of mortality and skeletal muscle dysfunction leading to mobility limitation and loss of functional independence, which can translate to poor quality of life [3,5,11].

Anabolic strategies such as exercise and oral nutritional supplementation (ONS) are proposed to improve MM and muscle quality and function in HD patients [12,13,14,15]. Indeed, several studies have shown that intradialytic ONS may improve nutritional status and reduce the risk of entering a catabolic state [12,13,16]. Furthermore, exercise has beneficial effects on MM, PF, energy intake, blood pressure, body weight, and quality of life (QOL) in some studies [17,18]. Exercise programs have been shown to improve different PF tests such as six-minute walk test (6 MWT), sit to stand test (STS5), time up and go (TUG) test among others [19,20,21,22]. However, there is still a significant debate about which type of exercise program is the best for dialysis patients and whether an exercise program with or without ONS provides superior benefits [23]. Regarding this last point, exercise without adequate nutritional supplementation in HD patients could be inadequate to promote protein anabolism [24], and it is well known that protein ingestion after a resistance exercise (RE) session increases protein synthesis and inhibits the breakdown of proteins in healthy subjects [25]. However, the combination of exercise and ONS has been poorly studied in HD patients. In previous studies, other researchers and our group evaluated the combination of exercise (either aerobic exercise (AE) or RE) and ONS, but the superiority of this strategy compared to ONS without exercise in MM was not observed [26,27,28,29,30]; in these previous studies we used 1 can of the ONS and we evaluated the effect of one type of exercise [26,28]. However, the novelty of this study is that we increased the ONS from 1 to 2 cans, combining it with AE and RE, and used a follow-up time from 3 months to 6 months. Based on these results, we conducted a pilot clinical trial to evaluate the effects of a 6-month intervention combining ONS with an intradialytic exercise program that combined both aerobic and resistance training on PF and MM quantity and quality (intramuscular lipid content via attenuation). We hypothesized that the combination of ONS with intradialytic aerobic and resistance exercise would enhance PF as well as the quantity and quality of MM in HD patients compared with ONS alone.

## 2. Materials and Methods

### 2.1. Study Design and Patients

This pilot clinical trial was conducted in accordance with the ethical standards set forth in the 1964 Declaration of Helsinki and in accordance with the Good Clinical Practice Principles of the International Conference on Harmonization. This study was approved by the ethics committees of our hospital with the registration number DI/18/105-B/04/021 and was registered with the clinical trial number ISRCTN63121006. Informed consent was obtained from all subjects involved in the study. The inclusion criteria were regular HD two or three times a week, age > 18 years, and ability to perform the PF tests. Patients with amputation, hospitalization in the last three months, unsatisfactory attendance at HD sessions, severe effort angina in accordance with the Canadian Cardiovascular Society (CCS level 3) or the New York Heart Association (NYHA stage 4) classification scale of heart failure, pregnancy, severe dyspnea, femoral fistula, arrhythmias, precordial pain, orthopedic or neurological compromises, or cognitive alterations affecting study participation were excluded. Additionally, patients with intolerance to ONS or intolerance/contraindications to the exercise routine, according to nephrologist and cardiologist evaluation, were excluded. After the intervention, possible confounders of the main outcome of physical activity were measured using the questionnaire of the University of Laval [31], the Charlson Comorbidity Index [32], and residual uresis.

### 2.2. Sample Size Calculation

To estimate the sample size, we calculated the effect size (F-value) for muscle quality according to the variable six-minute walk test [28]. Considering an effect size of f-value 0.38, an ANOVA was performed for repeated measures between factors, considering two intervention groups (difference between two independent means), two measurements (baseline and final), a correlation of 0.5 between measurements, and a power of 80% with a 95% confidence interval (*p* < 0.05). The sample size calculation was conducted with GPower 3.1^®^ (version 3.1.9.2; Heinrich-Heine-University, Düsseldorf, Germany). The minimum sample size was 20 subjects per group. Considering a loss to follow-up of 20%, we obtained a minimum sample size of 24 patients per group.

According to Wittes and Brittain et al., we included in this pilot study the 50% of the total sample size (24 patients) [33].

### 2.3. Intervention

All patients were provided a diet plan that was calculated based on the Kidney Disease Outcomes Quality Initiative guidelines [34]. Patients who met the inclusion criteria were randomized into one of the 2 groups using a block design with the Research Randomizer program (www.randomizer.org, accessed on 21 May 2019).

#### 2.3.1. Oral Nutritional Supplementation Group (ONS)

During the HD sessions, the patients received one can of a specialized oral nutritional supplement for dialysis patients that consisted of 434 kcal, 19.2 g protein, and 22.8 g lipids (Nepro with Carb Steady, Abbott Nutrition), and they received the other after their dialysis session to consume at home. To ensure that the patient consumed the second can, we asked for a photo of the empty can.

#### 2.3.2. Oral Nutritional Supplementation + Exercise Group (ONS + EX)

Patients in this group received the same two cans of the oral nutritional supplement. They drank one during the HD session while they were exercising and the other after dialysis to consume at home. Every session of exercise consisted of warm-up and cool-down phases in which patients cycled on a bike for 10 min without resistance and the rating of perceived exertion of the patients was very light according to the Borg scale (6–20) [35]. The conditioning phase consisted of a 6-month progressive and personalized exercise program that combined RE and AE. For AE, the time was established in the first session of exercise and was then gradually increased to reach 30 min. In the first session, patients were instructed to start cycling at a moderate intensity (somewhat hard: 12–13) according to the Borg scale of rating perceived exertion (RPE) without resistance. To calculate the resistance and time of the AE, every 5 min we showed the Borg scale to the patients to change or maintain the resistance of the bike, or we allowed the patients more time on the bicycle until reaching the desired intensity. After aerobic training, patients started the intradialytic RE routine. Patients were trained according to an adaptation of the program ‘‘Exercise: A Guide for People on Dialysis” [36]. Each subject used TheraBand Latex Resistance Bands^®^ to individualize the exercise; to decide the color of the band, patients started performing 10 repetitions of the exercise with the lowest resistance band, and the color was changed to other, harder colors if the intensity was not moderate according to the Borg scale (6–20). Four types of RE (lower leg extension, arm extension, straight leg extension, and seated marching) were performed during the HD session (4 sets × 20 repetitions). Details of the 4 types of exercise can be found elsewhere [28]. The exercise was individualized with the FITT principle (frequency, intensity, time, and type). At the end of all exercise sessions, patients provided us information related to the RPE and depending on every patient’s RPE the exercise was re-adjusted. The progression of RE consisted of increasing the ankle weights or the color of the resistance of the band, for AE, and time and resistance of the bicycle was also increased if the patient’s RPE was less than the target. The exercise intervention was administered and supervised by a trained dietitian (G.M.A) with experience in exercise programs for dialysis patients. For safety reasons, patients were never alone and heart rate and blood pressure were monitored during the exercise sessions.

### 2.4. Primary Outcomes

#### 2.4.1. Evaluation of the Quantity and Quality of Muscle Mass with Computed Tomography and Anthropometry

The evaluation of MM was performed by computed tomography (CT) and with anthropometry at baseline and at 6-month follow-up: (1) acquisition of images was carried out using 2 identical CT scanners (Siemens Somatom 128 slices, 2011), without the use of iodinated contrast, 2 times. Measurements of the muscle tissue were performed in workstations (Carestream Vue PACS) at the half of the femur in each patient. The protocol used was 0.8 mm slice thickness with a 3 mm reconstruction in a soft tissue window. CT scanner tube voltage was on average between 100 and 120 kV, exposure varied from 50 to 200 mAs, and a soft tissue Kernel was used. Muscle area and Muscle quality: a free hand ROI tool was used to draw the margins of the muscle tissue and aponeurosis to calculate the thigh muscle area (quantity of muscle mass) and intramuscular lipid content via attenuation (density values) also expressed in the average of Houndsfield units [37], and any incremental would express the substitution of fat tissue for muscle in the measured area. (2) Anthropometric measurements were taken with a Lange skinfold caliper by a trained dietitian (G.M.A.) before the HD sessions to estimate mid-arm muscle circumference and arm muscle area. To estimate both indicators of MM, we used the following formulas [38]:

- Mid-arm muscle circumference:
- Mid-arm circumference—(π × triceps skinfold thickness)
- Bone-free arm muscle area:
- Males = [(midarm circumference (cm) − π × triceps (cm)]^2^/4 π) − 10
- Females = [(midarm circumference (cm) − π × triceps (cm)]^2^/4 π) − 6.5

#### 2.4.2. Evaluation of Physical Function and Handgrip Strength

PF was assessed at baseline and at 3 and 6 months using the STS5, which measures the muscle strength of the lower limbs, and the short physical performance battery (SPPB) [39], which measures the global function of the patients. The STS5 measures the time taken to complete 5 repetitions of the sit-to-stand test. To perform this test, we used a chair with a height of 42 cm that was placed next to a wall. We asked patients to fold their arms across their chest and stand up and sit down five times as quickly as possible, and we took the time from the initial sitting position to the final standing position. The SPPB is a well-validated test and measures three different dimensions of the PF: 4 m gait speed, chair stand, and standing in three different positions for assessment of balance. Each of these tests was assigned a score ranging from 0 to 4, with 4 indicating the highest level of performance. Ultimately, we obtained a total score from 0 to 12, where the highest scores indicated better PF [39]. Other measurements of PF were the 6 MWT and TUG test; 6 MWT consisted of walking back and forth along a 22 m course (two 10 m straight lines connected by two 1 m curves) in a corridor for 6 min. We used the protocol of the American Thoracic Society [40].

Muscle strength was measured by hand dynamometry (Smedley III; Takei Scientific Instruments, Niigata City, Japan), whereby patients squeezed the dynamometer as hard as they could for 5 s. For patients who had a fistula, the measurement was performed with the hand opposite to the fistula; for patients with a catheter, the measurement was performed using the dominant hand. The measurement was taken three times, and the average of the three measurements was recorded as the handgrip strength.

### 2.5. Secondary Outcomes

#### 2.5.1. Body Composition and Nutritional Status Assessment

Body composition was measured using bioelectrical impedance 30 min after each HD session at baseline and at 24 weeks. The electrical properties of the body, such as resistance, reactance, and phase angle, were measured using multifrequency bioimpedance analysis (Seca 525 body composition analyzer). The nutritional status was evaluated using the malnutrition inflammation score (MIS) [41].

#### 2.5.2. Laboratory Parameter Assessment

Blood samples were taken before the HD session to determine measurements for creatinine, albumin, phosphorus, potassium, hemoglobin, and total lymphocyte count (TLC).

#### 2.5.3. Quality of Life Assessment

Quality of life was assessed at baseline and at 12 weeks using the Kidney Disease Quality of Life Short Form; this questionnaire assesses health-related concerns of individuals with kidney disease and on dialysis: symptoms/problems, effects of kidney disease on daily life, burden of kidney disease, work status, cognitive function, quality of social interaction, sexual function, and sleep. Each question was precoded numerically and was then transformed to values ranging from 0 to 100. Higher scores were associated with a better perception of QOL. Scores that were equal to or below the mean were indicators of lower QOL according to the standards of the Kidney Disease Quality of Life Short Form [42].

### 2.6. Statistical Methods

Categorical variables were reported as absolute numbers and proportions; Pearson’s chi-squared or Fisher’s exact tests were used to analyze changes. The primary analysis to assess the effects of the intervention was a repeated measure analysis of variance (2 groups × 3 time points). A secondary analysis was performed using repeated measured ANOVA or Friedman test according to the data distribution to perform comparisons in the same group. The effect size calculation was performed with Cohen’s d. This is a standardized effect size measurement based on standard deviation differences, with 0.2 considered a small effect, while 0.8 standard deviation is a large effect that could be a guide for clinical interpretation of the impact of a variable on an outcome of interest. We performed multivariate regression analysis to estimate the possible effects of potentially confounding variables such as age, sex, and comorbidities on physical functionality parameters by calculating the standardized beta coefficient, confidence intervals (CI), and *p*-value. *p* < 0.05 and 95% confidence interval were considered statistically significant. SPSS version 21.0 was used to analyze the data.

## 3. Results

**Figure 1:**
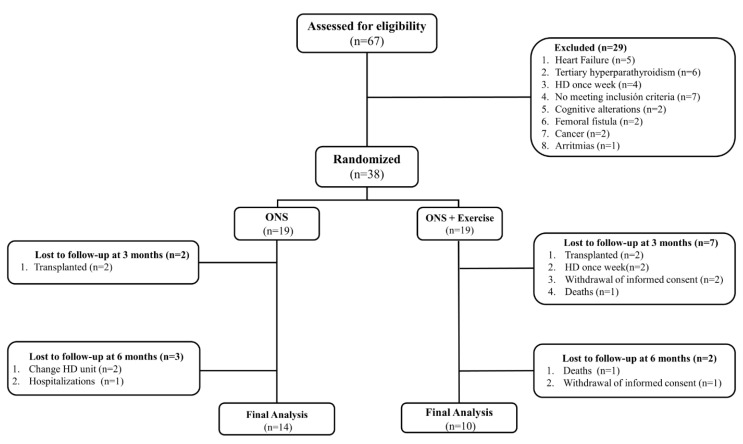
Sreening, randomization and follow-up according to the CONSORT diagram.

All the patients in our HD unit (*n* = 67) were assessed for eligibility by a nephrologist according to the inclusion and exclusion criteria; of these, 38 patients were included and randomized. At the end of the study, 24 patients were analyzed (Figure 1).

### 3.1. Baseline Characteristics

At the beginning of the study, no statistically significant differences were observed in any of the variables. The median age of the patients was 34 ± 11 years, 10 (41.7%) of the patients were male, and the etiology of CKD was unknown in most cases (66.7%). Most of our population received HD sessions twice a week (87.5%) (Table 1).

### 3.2. Changes in the Quality and Quantity of Muscle Mass Measured with Computed Tomography and Anthropometry

The thigh muscle area and the quality of muscle mass (intramuscular lipid content via attenuation) did not show significant change in either group. In the intergroup comparison, there was a trend for an improvement in muscle quality in the ONS + EX group compared with ONS alone, though this difference was not statistically significant (ONS, Δ −1.1 HU vs. ONS + EX, Δ −1.5 HU; *p* = 0.054). Regarding the anthropometric indicators of MM, no significant increases were found at the end of the intervention (Table 2). Multivariate regression analyses revealed that sex had a significant influence on tight muscle area (standardized beta coefficient = −0.601 (CI −35.72–−6.48), *p* = 0.007) while age showed a similar effect on quality of muscle mass (standardized beta coefficient = −0.491 (CI −0.31–−0.01), *p* = 0.029) in volunteers of the ONS + EX group.

### 3.3. Changes in the Physical Function Tests and Handgrip Strength

**Figure 2:**
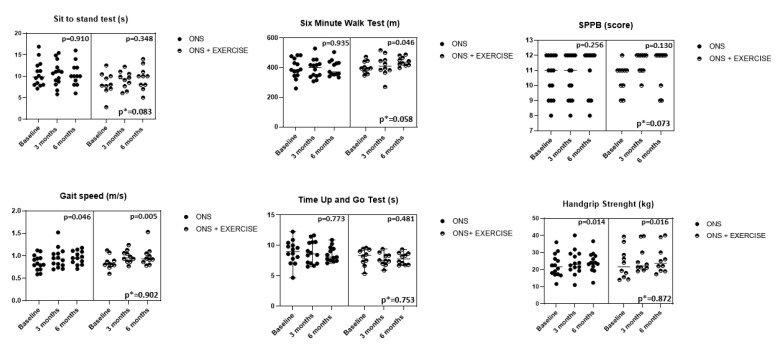
Intragroup and intergroup changes in physical function tests. p*: ANOVA (3 times × 2 groups).

The ONS group showed intragroup improvements in gait speed (*p* = 0.046) and HGS (*p* = 0.014), while the ONS + EX group showed intragroup improvements in gait speed (*p* = 0.005), 6 MWT (*p* = 0.046), and HGS (*p* = 0.016). No statistically significance differences were observed between groups; however, there were trends for improvements in the 6 MWT (*p* = 0.058) and SPPB score (*p* = 0.073) in ONS + EX compared with ONS alone (Figure 2). Multivariate regression analyses confirmed that potentially confounding variables such as sex or age did not significantly influence gait speed, HGS, and 6 MWT in the ONS or ONS + EX groups. We also observed greater effect sizes in the 6 MWT and TUG test in favor of the exercise group (Table 3).

### 3.4. Secondary Outcomes

Regarding body composition indicators, both groups exhibited significant increases in body weight, but no difference was found between the two groups. The ONS + EX increased the percentage of fat mass (*p* = 0.046). Both groups also had nonsignificant increases in nutritional status, as evaluated by MIS score. Regarding biochemical parameters, the ONS group had a statistically significant decrease in the serum concentrations of creatinine (*p* = 0.049) but no changes were observed in the other variables. No statistically significant differences were observed in the intergroup and intragroup comparisons in the bioelectrical impedance analysis parameters of resistance, reactance, and phase angle (Table 2).

Finally, for QOL, we observed (Table 4) significant improvements in the ONS group for two items of the specific part (symptoms and burden of kidney disease) and in one item of the generic part (social function). In the ONS + EX group, we observed significant improvements in the emotional well-being item and in the emotional role item. We performed multivariate regression analyses and found no significant effects of potentially confounding variables on secondary outcomes.

## 4. Discussion

In this pilot clinical trial, we compared the effects of ONS versus the combination of ONS with intradialytic aerobic and resistance training on PF and the quality and quantity of the MM over a six-month period.

The primary findings of this study include the following: (1) several measures of physical function improved in both groups, with a trend for greater improvements in the 6 MWT and TUG test in ONS + EX compared with ONS alone; (2) while MM quantity did not improve in either group, there was a trend for an improvement in muscle quality in ONS + EX compared with ONS alone; (3) there were no intergroup differences in QOL changes. Taken together, these data suggest that the combination of ONS with intradialytic aerobic and resistance training has modest benefits compared with ONS alone.

We observed that ONS alone improves PF as measured by gait speed and HGS. Regarding these results, our group has shown in two previous studies that ONS without exercise for three months during HD sessions had a positive and significant impact on muscle strength, TUG test, and the 6 MWT, although it should be noted that the largest effect sizes were observed when nutritional supplementation was combined with exercise [26,27,28]. Another study that reported significant results for PF measured by gait speed after 3 and 6 months in the ONS group was the IHOPE study carried out by Jeong et al. [29], where significant increases of 12% and 13% were observed at three and six months of follow-up, respectively.

In relation to weight gain and nutritional status, the ONS group significantly increased body weight but no statistically significant improvements in the MIS score were observed. Similar to these findings, Ramos-Acevedo et al. [43] recently reported that after three months of intervention with intradialytic ONS, the dry weight improved significantly and contrary to our findings, in this study MIS score improved significantly.

No gain in MM measured with CT or anthropometric indicators was observed in the group that received ONS alone, which has been evidenced in other studies; van Vliet et al. [44] reported that ingesting a meal rich in kilocalories and protein on a different day of the HD session did not stimulate muscle protein synthesis. With these results, we could infer that this intervention is insufficient to achieve a significant gain in MM [44].

Another strategy that has been shown to have a positive impact on variables such as physical and muscular performance is exercise. The effects of exercise in HD patients have been shown to improve variables such as muscle strength, blood pressure, QoL [17,18], and different PF tests such as gait speed, STS5, and TUG test [18,19,21,22]. However, according to Johansen et al. [24], performing exercise without adequate nutritional support may not improve muscle anabolism, and it is also well known that the ingestion of protein after an exercise session increases the synthesis of proteins and inhibits muscle proteolysis [24].

The combination of exercise with ONS has been scarcely studied in HD patients. In this study, ONS + EX showed intragroup differences in gait speed, in 6 MWT, and HGS but no differences were observed between groups; however, greater effect sizes in favor of the ONS + EX group were observed in the 6 MWT and TUG test.

In previous studies, our group showed very similar results; in our first randomized clinical trial, patients exercised at a moderate intensity for 12 weeks, they performed 4 intradialytic RE, and they received one can of Nepro HP (Abbott), but there were no significant differences in nutritional status, MM, or HGS compared to ONS alone [26].

In the AVANTE-HEMO study, we randomized patients into three different groups: (1) ONS group; (2) ONS + RE; and (3) ONS + AE, after 12 weeks. No differences were observed in PF tests, but we reported greater effects sizes in the different PF tests in the EX-group. Again, we did not observe differences in the intra group or intergroup comparisons in MM [28].

Similar to our findings, Dong et al., in a study of 6 months, reported no significant differences between the group that exercised compared to ONS alone in the one repetition maximum (1-RM) leg strength, body weight or lean mass measured with DEXA, but the volume of exercise was low (3 sets of 12 repetitions of leg press at an intensity of 70%, 3 days per week) [27].

In one of the largest studies, Jeong et al. showed that before 12 months of intradialytic AE (30–45 min at a moderate intensity) + whey protein (30 g), no significant differences were observed between the control group or the group that received the whey protein alone in the primary outcome (shuttle walk test) [29].

None of the clinical trials that have evaluated this combined strategy have shown significant increases in the size of MM [27,29,30]. Molsted et al. [45] showed that high-load strength training is associated with improvements in muscle strength and power, physical performance, and QOL but no significant increases were observed in muscle hypertrophy. For improving muscle hypertrophy in older patients, the American College of Sports Medicine (ACSM) recommends progressive resistance training prescription that control different variables such as muscle activation, type of strength exercise, order of exercise, training load, volume and rest [46]. This includes performing eccentric, concentric, monoarticular, and multiarticular exercises two to three times a week, working first on higher intensity exercises with loads of 60% to 70% of the 1-RM and performing one to three sets of eight to twelve repetitions with rests of 1 to 2 min in between [46]. Despite the above, a wide variety of specific ranges of muscle mass gain have been reported in the literature, and there is no consensus on the best training program for hypertrophy [47]. Wider ranges and training options are currently recommended, where low exercise intensities (30–60% of 1-RM) have been shown to have the same effect as training performed at more than 60% of 1-RM [47].

While our exercise program was designed to increase muscle size and strength, the excessive muscle catabolism in HD patients may have been responsible for the modest benefits that we saw. Because of this, it may be necessary to consider more aggressive strategies to better control muscle catabolism [48,49]. Johansen et al. [24] showed that the only groups that had a significant gain in lean mass were the groups that received the anabolic steroid independent of carrying out the exercise while the exercise group without the steroid increase fat mass and decrease lean mass. Based on the above, novel strategies, such as testosterone, vitamin D, growth hormone, and myostatin inhibitors are beginning to be studied to stop the loss of MM [1]. In experimental models, Zhang et al. [49] showed that after 4 weeks of pharmacological inhibition of the protein myostatin, the loss of body weight and muscle mass in rats with CKD was reversed.

Despite the null impact of exercise on the amount of MM, there was a trend for an improvement in muscle quality in the exercise group compared with the group that received ONS alone. These findings are in line with the results reported by Cheema et al. [22], who also found that 12 weeks of resistance training in HD patients improves muscle quality, but not muscle mass. Similar findings have also been found in older adults, where it has been shown that 24 weeks of resistance training improved muscle quantity and quality [50].

Some of the limitations to the study were the high rate of loss to follow-up; however, in previous interventions, our group reported similar losses. Another limitation in this study was the good physical function our patients had at baseline. Some of the factors that limit the external validity of the study findings are that most of our patients received suboptimal doses of dialysis, and our population is younger than most others in the published literature. Another limitation is that the intensity of the exercise was not evaluated with objective tools such as 1RM for RE training or heart rate for AE. Last, although multivariate analyses indicated that most confounding variables did not significantly influence the improvements in gait speed, HGS, and 6 MWT in the ONS + EX group, we found that sex and age significantly influenced tight muscle area and quality of muscle mass, respectively. For this reason, we are working on increasing the number of volunteers in either ONS or ONS + EX groups to amend these limitations and draw more accurate conclusions.

## 5. Conclusions

Exercise in combination with ONS does not improve the quantity of MM compared to ONS without exercise; however, modest improvements could be observed in more PF variables and quality of MM in the group that performed exercise. It is possible that exercise is insufficient to block the multiple catabolic mechanisms that lead patients to have progressive decreases in MM. [51]. Future clinical trials should be designed to study novel strategies that may improve both the quantity and quality of MM.

## Figures and Tables

**Table 1:** Demographics and baseline characteristics.

Variables	ONS (n = 14)	ONS + EXERCISE (n = 10)	*p*
Age (years) mean ± SD	38.14 ± 12	28.5 ± 9.5	0.047
Male (n/%)	5 (35.7)	5 (50)	0.484
Etiology (n/%)			0.318
Unknown	9 (64.3)	7 (70)	
Diabetes mellitus	2 (14.3)	0 (0)	
Glomerulopathy	1 (7.1)	0 (0)	
Hypertension	1 (7.1)	3 (30)	
Other	1 (7.1)	0 (0)	
Frequency of dialysis (%)			0.550
2 times per week	13 (92.9)	8 (80)	
3 times per week	1 (7.1)	2 (20)	
Dialysis vintage, months	61 ± 43	33 ± 19	0.078
Comorbidities (%)			
Diabetes	2 (14.3)	0 (0)	0.493
Hypertension	14 (100)	10 (100)	0.05
Vascular access (%)			0.188
Catheter	8 (57.1)	7 (70)	
AV fistula	6 (42.9)	3 (30)	
Residual uresis (ml)	0 (0.0–162)	0 (0.0–0.0)	0.546
Charlson Index Comorbidity	2 (2–4)	2 (2–2)	0.259

Data are indicated as absolute number (percentage), mean ± SD, or median. ONS, oral nutritional supplementation; BMI, body mass index; MAC, mid-arm circumference; AMC, arm muscle circumference; AMA, arm muscle area; FM%, fat mass as a percentage of body weight; MIS; malnutrition inflammation score; PAQ, physical activity questionnaire.

**Table 2:** Changes in anthropometrics, nutrition status, body composition, and blood chemistry.

Variables	ONS (n = 14)			ONS + EXERCISE (n = 10)			
	BASELINE (n = 14)	6 MONTHS (n = 14)	p*	BASELINE (n = 10)	6 MONTHS (n = 10)	p*	*p+*
Anthropometrics Weight (kg) Mid-arm circumference (cm) Arm muscle circumference (mm) Arm muscle area (cm^2^) Fat mass (%) Triceps skin-fold thickness (mm)	54.7 ± 7.4 27 ± 3.1 230 (213–249) 37 ± 8.8 23 ± 8.4 12.8 ± 4.6	55.8 ± 6.7 26 ± 3 220 (207–238) 33.9 ± 9.1 23.8 ± 8.2 13.1 ± 5.2	0.014 0.151 0.084 0.097 0.311 0.537	56.2 ± 8.8 27.1 ± 3.5 228 (209–257) 36 ± 9.8 21.1 ± 7 13 ± 5.1	58.2 ± 9.2 26.9 ± 3.1 226 (207–246) 34.7 ± 9.2 22.9 ± 7.9 13.7 ± 5.2	0.001 0.778 0.508 0.544 0.046 0.066	0.462 0.770 0.886 0.838 0.793 0.798
MIS	5.5 (3.7–8.0)	5 (3.5–8)	0.063	4 (3–6.5)	3.5 (1.7–6)	0.086	0.259
Bioimpedance analysis Resistance (ohm) Reactance (ohm) Phase angle (°)	593 ± 96 57 ± 12 5.5 ± 0.98	599 ± 118 59 ± 21 5.5 ± 1.5	0.750 0.651 0.896	631 ± 109 64 ± 14 5.8 ± 0.68	622 ± 109 60 ± 13 5.5 ± 1.1	0.586 0.443 0.515	0.633 0.876 0.992
Computed tomography Muscle attenuation (HU) Thigh muscle area (cm^2^)	52 ± 5.3 96.2 ± 24	53 ± 3.7 98 ± 20	0.592 0.138	54.6 ± 3.4 100 ± 14	56 ± 3.3 97 ± 12	0.280 0.205	0.054 0.895
Biochemical parameters Hemoglobin (g/dL) Total lymphocytes count (cells/mm^3^) Creatinine (mg/dL) Albumin (g/dL) Phosphorus (mg/dL) Potassium (mmol/L) CRP (mg/L)	9.8 ± 1.8 1013 (850–1313) 13.3 ± 2.8 4.3 ± 0.41 5.9 ± 2.3 5.7 (5–6.1) 5.6 (2.8–8.9)	9.9 ± 1.4 886 (795–1263) 11.4 ± 4.4 4.3 ± 0.47 5.2 ± 2.2 5.4 (5–5.8) 4.1 (2–7.3)	0.834 0.551 0.049 0.390 0.128 0.115 0.638	10.9 ± 2 1065 (932–1556) 13.3 ± 3.5 4.2 ± 0.53 6.1 ± 2.1 5.1 (4.6–6.1) 4.5 (1.2–12.8)	10.6 ± 1.8 1038 (864–1240) 13.5 ± 2.2 4.2 ± 0.29 5.5 ± 1.5 4.9 (4.7–5.6) 3.3 (2.9–9)	0.740 0.594 0.873 0.849 0.242 0.212 0.594	0.306 0.477 0.207 0.396 0.770 0.336 0.781

Data are represented as mean ± standard deviations. ONS, oral nutritional supplementation; MIS, malnutrition inflammation score. p* Student *t* test or Wilcoxon to compare intragroup differences. *p*+ Student t test or U Mann–Whitney to compare intergroup differences.

**Table 3:** Effect size (Cohen’s-d) calculation for physical function tests.

Variables	Cohen’s-d
Six-minute walk test (m)	1.02
Gait speed (m/s)	0.17
5-Sit to stand test (s)	0.33
Timed up and go test (s)	0.63
Handgrip strength (kg)	0.30
SPPB (score)	0.07

Cohen’s-d was calculated considering two groups. ONS, oral nutritional supplementation; ONS + EX; oral nutritional supplementation plus exercise.

**Table 4:** Changes in the quality of life measured with KDQOL SF-26.

	ONS (n = 14)			ONS + EXERCISE (n = 10)		
Specific part	Pre	Post	p*	Pre	Post	p*
Symptoms	74.1 ± 11.9	82.4 ± 9.8	0.04	83.5 ± 6.1	86.1 ± 7.9	0.25
Effects of Kidney disease	61.7 ± 21.3	73 ± 25	0.15	74.1 ± 12.3	74.4 ± 22	0.94
Burden of kidney disease	47.3 ± 15.6	59.3 ± 18.7	0.00	63 ± 14.7	57.8 ± 16.8	0.28
Work status	41.6 ± 41.7	50 ± 42.6	0.50	66.6 ± 38.9	62.5 ± 48.2	0.80
Cognitive function	25.5 ± 17.2	26.6 ± 17.9	0.85	15.5 ± 11.8	12.2 ± 9.7	0.35
Quality of social interaction	33.3 ± 15.8	27.7 ± 13.5	0.31	14.4 ± 9.7	19 ± 18	0.47
Sexual function	83.3 ± 28.8	75 ± 43.3	0.42	78.1 ± 31.1	65.6 ± 37.6	0.22
Sleep	66.8 ± 21.1	71.6 ± 13.7	0.42	78.7 ± 8.8	83.3 ± 13	0.17
Social Support	62.4 ± 18.9	70.8 ± 16	0.13	66.6 ± 14.2	66.6 ± 25.6	1.00
Dialysis staff encouragement	77 ± 11.7	73.9 ± 8.3	0.38	73.9 ± 11.2	77 ± 4.8	0.33
Patient satisfaction	74.2 ± 17.2	68.1 ± 26.3	0.22	72.2 ± 16.4	68 ± 22.9	0.51
Generic part	Pre	Post	p*	Pre	Post	p*
Physical function	74.1 ± 15.6	75.4 ± 20.6	0.78	88.3 ± 8.3	86.2 ± 7.4	0.21
Physical role	56.2 ± 44.1	56.2 ± 44.1	1.00	87.5 ± 31	85.4 ± 34.4	0.79
Pain	85.6 ± 16.1	85.2 ± 21.8	0.94	74.7 ± 28.3	79.1 ± 30.4	0.61
General Health perceptions	40.4 ± 13.8	46.6 ± 13.4	0.20	61.2 ± 9.5	58.7 ± 15.9	0.54
Emotional well-being	68.3 ± 18	73 ± 22.1	0.48	75.3 ± 16.2	82 ± 13.9	0.08
Emotional role	66.6 ± 34.8	66.6 ± 34.8	1.00	69.4 ± 36.1	97.2 ± 9.6	0.02
Social function	75 ± 25.5	94.7 ± 14.5	0.01	88.5 ± 13.5	86.4 ± 20.9	0.74
Energy/fatigue	61.6 ± 16.2	64.5 ± 18.6	0.58	70.8 ± 14.5	70.4 ± 18.1	0.94

Data are represented as mean ± standard deviations. ONS, oral nutritional supplementation. p*: student t test for related samples.

## Data Availability

Datasets analyzed or generated during the study can be found at: ISRCTN—ISRCTN63121006: Effect of nutritional supplement taken with and without exercise on muscle condition in adults on hemodialysis: A randomized controlled trial.
